# Time-dependent probabilistic tsunami risk assessment: application to Tofino, British Columbia, Canada, subjected to Cascadia subduction earthquakes

**DOI:** 10.1038/s44304-024-00006-x

**Published:** 2024-05-07

**Authors:** Katsuichiro Goda, Raffaele De Risi

**Affiliations:** 1 Department of Earth Sciences, Western University, London, ON Canada; 2 Department of Statistical & Actuarial Sciences, Western University, London, ON Canada; 3 Department of Civil Engineering, University of Bristol, Bristol, UK

**Keywords:** Natural hazards, Ocean sciences, Solid Earth sciences

## Abstract

A new time-dependent probabilistic tsunami risk model is developed to facilitate the long-term risk management strategies for coastal communities. The model incorporates the time-dependency of earthquake occurrence and considers numerous heterogeneous slip distributions via a stochastic source modeling approach. Tidal level effects are examined by considering different baseline sea levels. The model is applied to Tofino, British Columbia, Canada within the Cascadia subduction zone. High-resolution topography and high-quality exposure data are utilized to accurately evaluate tsunami damage and economic loss to buildings. The results are tsunami loss curves accounting for different elapsed times since the last major event. The evolutionary aspects of Tofino’s time-dependent tsunami risk profiles show that the current tsunami risk is lower than the tsunami risk based on the conventional time-independent Poisson occurrence model. In contrast, the future tsunami risk in 2100 will exceed the time-independent tsunami risk estimate.

## Introduction

Coastal communities within major subduction regions face risks dominated by low probability and high consequences of mega-tsunamis^1^. Quantitative tsunami risk assessments are instrumental in developing risk-based disaster management strategies for municipalities, governments, and financial institutions^2,3^. Conventionally, worst-case historical scenarios were adopted to create tsunami risk mitigation plans for safeguarding people and assets in coastal areas. Catastrophic tsunami events in the Indian Ocean and Japan highlighted that historical data alone are insufficient to define extreme scenarios^4^. Since then, probabilistic approaches have become popular and have been used to complement deterministic scenario-based approaches^5–7^. A typical output of probabilistic tsunami risk analysis consists of an exceedance probability (EP) curve, displaying aggregate tsunami loss to individuals or organizations as a function of the exceedance probability^8^. From the EP curve, various risk metrics, such as average annual loss (AAL) and value at risk (VaR), can be derived and used to make informed disaster risk management decisions.

Probabilistic tsunami hazard and risk assessments allow explicit and rigorous treatment of uncertainties associated with tsunami sources and tsunami impacts on the built environment^9^. The key influential elements of these analyses include earthquake occurrence, magnitude-frequency relationship, rupture process, tsunami generation and propagation, tsunami run-up and inundation, tsunami vulnerability, and tsunami loss estimation^10,11^. Recent advances in probabilistic tsunami hazard and risk analysis methods include the consideration of time-dependent renewal models for earthquake occurrence^12,13^, the consideration of heterogeneous earthquake slips with variable fault-plane geometry^14,15^, the implementation of a logic tree to consider multiple alternatives of the models and parameters^16,17^, and the development of tsunami fragility functions based on an extensive tsunami damage dataset^18,19^. Despite these refinements, it is not straightforward to perform time-dependent probabilistic tsunami risk assessments of a coastal community based on numerous earthquake rupture scenarios (e.g., thousands), high-resolution topographic data (e.g., grid resolution less than 10 m), and high-quality building exposure data (e.g., building-by-building surveys). All the elements mentioned above are critically important for assessing quantitative tsunami risks accurately.

**Fig. 1:**
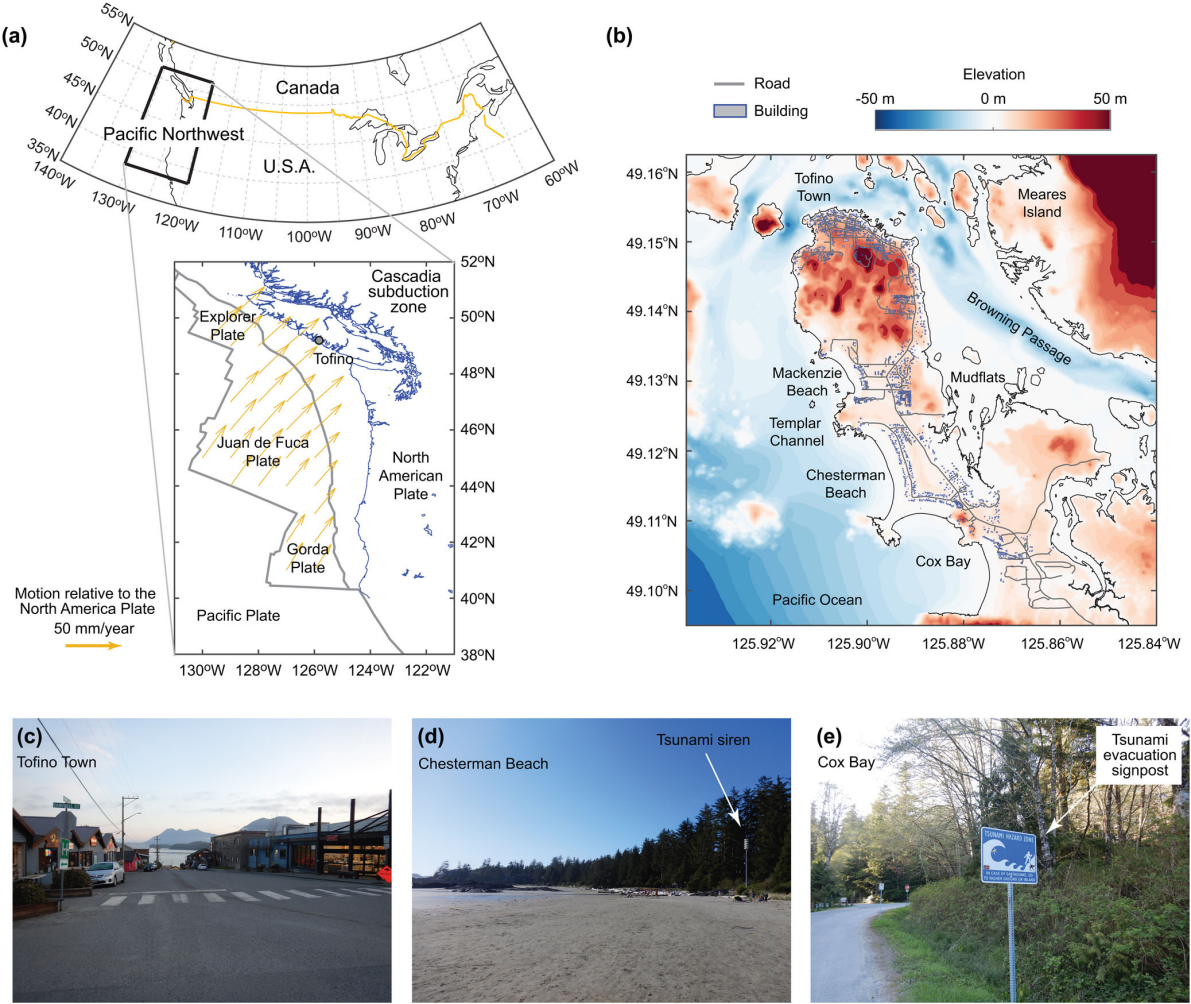
The District of Tofino and its surrounding environments. **a** Seismotectonic environment of the Cascadia subduction region, **b** elevation map together with buildings and roads in Tofino, **c** photo in Tofino Town, **d** photo at Chesterman Beach, and **e** photo at Cox Bay.

This study presents a new probabilistic tsunami risk assessment for a Canadian coastal community facing significant tsunami threats from the Cascadia subduction zone in the Pacific Northwest^20,21^. The seismic hazards in the Cascadia subduction region are driven by the thrusting movements of the Juan de Fuca, Gorda, and Explorer Plates, which subduct underneath the North American Plate (Fig. 1a). The Cascadia subduction zone spans from Vancouver Island to northern California (circa 1,100 km). It hosted moment magnitude (*M*_w_) 9-class earthquakes in the past, which is supported by onshore and offshore geological evidence^22,23^, while the most recent earthquake occurred in 1700^24^. Past studies of the tsunami hazard assessments for Canadian coastal communities have advanced by considering a small number of earthquake rupture scenarios, for instance, five scenarios with variable earthquake magnitudes^25^ and five splay-fault/trench-breaching scenarios^26^. A comprehensive set of 5,000 stochastic source models for the partial and whole rupture patterns with magnitudes between *M*_w_ 8.1 and *M*_w_ 9.1 has been recently developed^27^. Subsequently, regional probabilistic tsunami hazard analysis for coastal locations along Vancouver Island was conducted^28^ by taking into account stochastic source models^27^ and the new time-dependent earthquake occurrence model for the full-rupture Cascadia subduction earthquakes^29^. The newly developed earthquake occurrence model accounts for uncertainty associated with radiocarbon dating of offshore geological records^30^ and allows three modes of possible earthquake recurrences in the Cascadia subduction zone using the Gaussian mixture model. Despite this, the analyses^28^ were limited to offshore locations at a regional scale and lacked high-resolution tsunami inundation simulations (i.e., grid resolution of 270 m). Although detailed building exposure data were used for tsunami risk assessments in the coastal town of Tofino^31^, a whole tsunami risk assessment by considering the time-dependent earthquake occurrence model, a comprehensive set of stochastic source models, high-resolution tsunami inundation simulations, and high-quality building exposure data, has not been conducted to date. This paper performs a time-dependent probabilistic tsunami risk assessment for Tofino using the latest hazard-exposure-vulnerability models, constituting a significant research innovation. A focus is given to the evolutional aspects of the tsunami risk due to the increasing time since the last full-rupture event in 1700. Therefore, the tsunami risk assessments can be continuously updated with the elapsed time. The results will be discussed in terms of EP curves, inundation maps, tsunami loss maps, and risk metrics (e.g., AAL and VaR) for different probabilities of exceedance. The developed tsunami risk model opens a new avenue for conducting a long-term tsunami risk assessment for coastal communities and informing their long-term risk management strategies.

## Results

### The physical environment of Tofino

Tofino is located at Esowista Peninsula within Clayoquot Sound on Vancouver Island (Fig. 1b) and is famous for sandy beaches, inlets, and ancient rainforests. The population in Tofino is 2516 (2021 Census), while in the summer peak times, 5000 to 8000 visitors and seasonal workers arrive and stay there. The main commercial area of Tofino is at the tip of the peninsula and is at a high elevation (above 10 m); thus, the tsunami risk in Tofino Town is relatively low (Fig. 1c). On the other hand, many houses and touristic facilities, such as resort hotels and campsites, are located in beach areas where the elevations are below 10 m (Fig. 1b), thereby the tsunami risk is high in these areas. The beach areas are equipped with a siren system for tsunami warnings (Fig. 1d) and tsunami evacuation signages (Fig. 1e). Earthquake and tsunami hazards from the Cascadia subduction zone^32^ are major threats for Tofino. The changing environmental condition due to the relative sea level rise in Clayoquot Sound, which could reach + 0.7 m by 2100, compared with the mean sea level in 2000^33^, will intensify storms and coastal floods as well as tsunamis in the future. A tidal monitoring station near Tofino Town has been operational since 1909; the tidal levels vary between –2 m and +2 m with respect to the long-term average tidal level.

To perform earthquake-tsunami and coastal hazard and risk assessments based on high-resolution bathymetry-elevation data, the District of Tofino conducted bathymetric surveys in the surrounding shallow water areas and LiDAR inland surveys. Tofino also developed a comprehensive inventory of buildings, cultural/historical sites, water sanitation facilities, potential environmental contamination sites, and infrastructures. As part of this exposure data development, a building-by-building inspection was conducted to determine critical structural features of the buildings (e.g., material, number of stories, and construction years). The building inventory includes 1,789 structures for residential, commercial, industrial, and civic occupancy (Fig. 1b), with a total asset value of 2.27 billion Canadian dollars (C$). These available data are employed in developing a tsunami risk model for Tofino (see the **Method** section).

### Time-dependent tsunami risk model

**Fig. 2:**
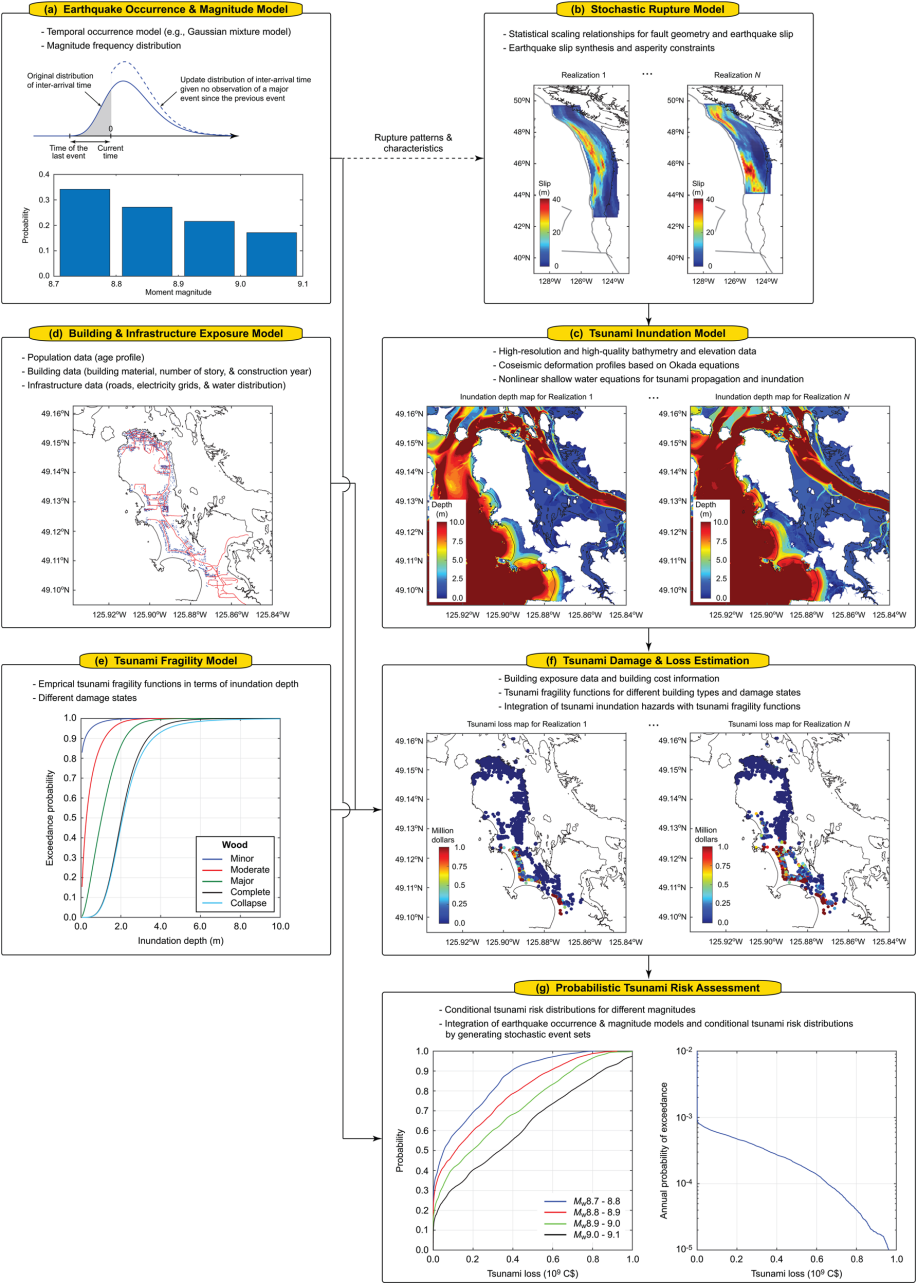
Time-dependent probabilistic tsunami risk assessment framework. **a** Earthquake occurrence and magnitude model, **b** stochastic rupture model, **c** tsunami inundation model, **d** building and infrastructure exposure model, **e** tsunami fragility model, **f** tsunami damage and loss estimation, and **g** probabilistic tsunami risk assessment.

The time-dependent probabilistic tsunami risk model is developed for Tofino by focusing on the Cascadia subduction earthquakes as the primary tsunamigenic sources. A computational framework of the tsunami risk model is shown in Fig. 2. The framework adopts a catastrophe modeling approach^8^ by integrating the earthquake occurrence and magnitude model, stochastic source model, tsunami inundation model, building and infrastructure exposure model, tsunami fragility model, tsunami damage and loss estimation, and probabilistic tsunami risk assessment^10^. Details of the model components are described in the Method section.

**Fig. 3:**
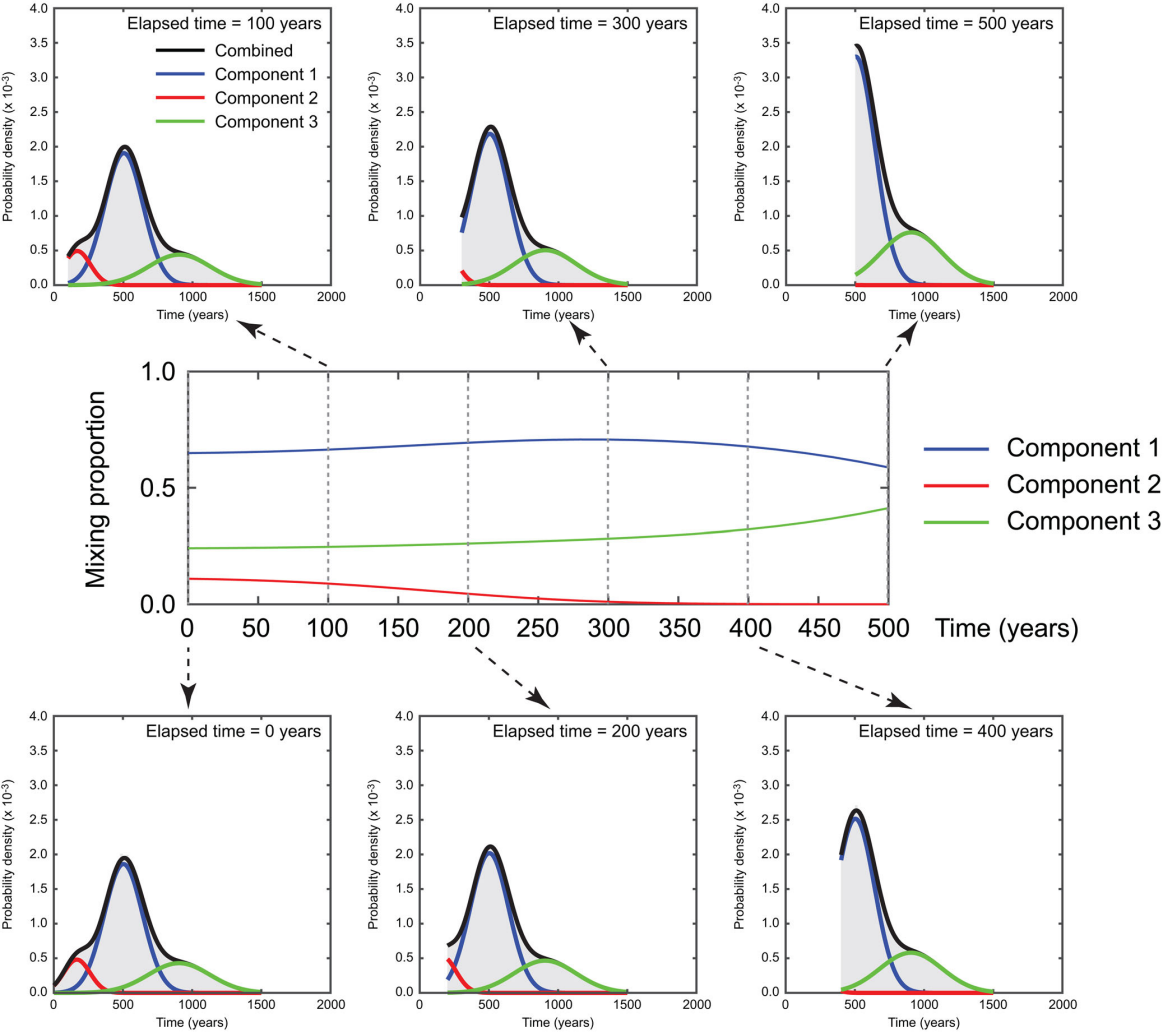
Time-dependent occurrence of the full-margin Cascadia subduction earthquakes. The mixing proportions for the Gaussian mixture model are shown in the middle panel over the elapsed time since the last major event. The conditional probability distributions of the occurrence time for different elapsed times ranging from 0 years to 500 years are shown in the bottom and upper panels.

Among the key elements, earthquake occurrence modeling is crucial (Fig. 2a) but involves significant uncertainty. This study focuses on the evolutionary aspects of quantified tsunami risks to the buildings in Tofino by considering the time that has elapsed since the last major event. Specifically, this study adopts a 3-component Gaussian mixture model^29^ to characterize the inter-arrival times of successive Cascadia megathrust events. The main component corresponds to earthquake recurrence with mean (μ) = 503 years and standard deviation (σ) = 139 years with a mixing proportion of 0.646. The second component represents long gaps (μ = 905 years and σ = 224 years) with a mixing proportion of 0.240, whereas the third component represents short-term clustering (μ = 167 years and σ = 95 years) with a mixing proportion of 0.114. The mixing proportions and Gaussian parameters (i.e., mean and standard deviation) of the three components are simultaneously estimated via the Expectation-Maximization algorithm^29^. The above-mentioned mixing proportions for the three components correspond to the elapsed time of 0 years (i.e., immediately after a major event). With the increasing time elapsed since the last major event, the mixing proportion for the short-term clustering decreases, while those for the intermediate and long-term clustering increase gradually over time. When the elapsed time becomes very long (exceeding the mean recurrence period), the mixing proportions for the short-term and intermediate clustering decrease, while that for the long-term clustering increases. Such evolutionary earthquake occurrence of the Cascadia subduction earthquakes is illustrated in Fig. 3. The middle panel shows the temporal variations of the mixing proportions of the three components. In the top and bottom-row panels, the conditional probability distributions of the inter-arrival time of the Cascadia subduction events at the elapsed times of 0, 100, 200, 300, 400, and 500 years are displayed by distinguishing the three components with different colors.

### Tsunami loss curves and tsunami inundation-loss maps

**Fig. 4:**
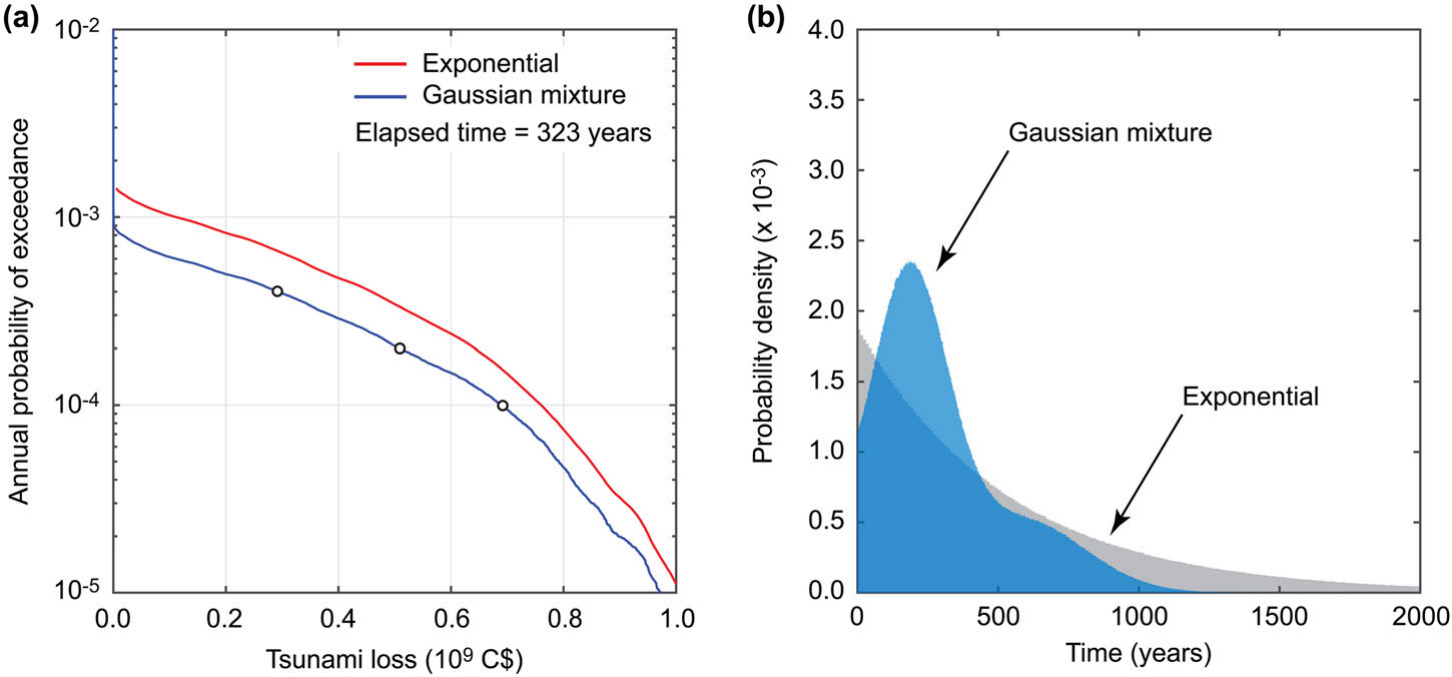
Effects of earthquake occurrence models on estimated tsunami loss in Tofino. **a** Exceedance probability tsunami loss curves for the time-dependent (Gaussian mixture) and time-independent (exponential) earthquake occurrence cases. **b** Probability distributions of earthquake occurrence time for the time-dependent and time-independent hazard cases. The three circles on the tsunami loss curve for the Gaussian mixture case correspond to the annual probability of exceedance of 0.0004, 0.0002, and 0.0001, for which the tsunami inundation and loss maps are shown in Fig. 5.

Using the developed tsunami risk model for Tofino, a current EP tsunami loss curve for the time-dependent earthquake hazards with the elapsed time of 323 years (i.e., Year 2023) is obtained and shown in Fig. 4a. As a benchmark, the tsunami loss curve for the time-independent earthquake hazards (i.e., inter-arrival time is exponentially distributed with a mean recurrence period of 561 years) is also included in the figure. These assessments set the tidal level to the mean sea level. An important observation from the current time-dependent tsunami loss curve shown in Fig. 4a is that tsunami loss occurrence is rare even for Tofino, one of the most exposed coastal communities to the Cascadia subduction event. Unlike tsunami hazard curves for wave amplitude or run-up, such a tsunami risk curve accounts for the spatial distribution of the buildings and their vulnerability. Due to frequent storms, houses adjacent to the beaches are equipped with some coastal protection (elevated grounds and stone fences). Therefore, moderate coastal flooding does not usually result in major loss.

A comparison of the time-dependent and time-independent tsunami loss curves indicates that the conventional Poisson process assumption results in the overestimation of the tsunami risk for Tofino. This is because the probability of having the next Cascadia subduction event within one year based on the Gaussian mixture model is smaller than the counterpart probability based on the exponential model. To show this more clearly, Fig. 4b compares the probability distributions of the earthquake occurrence time for the two cases. The horizontal axis is the time measured with respect to the current elapsed time. In this figure, the probability density value of the exponential model at time 0 is higher than that of the Gaussian mixture model. As inspected in Fig. 3, with the progress of the time without observing the next Cascadia subduction event, the conditional probability distribution of the earthquake occurrence time for the Gaussian mixture model will take a greater value at time 0. In contrast, the probability distribution of the exponential model will remain unchanged.

**Fig. 5:**
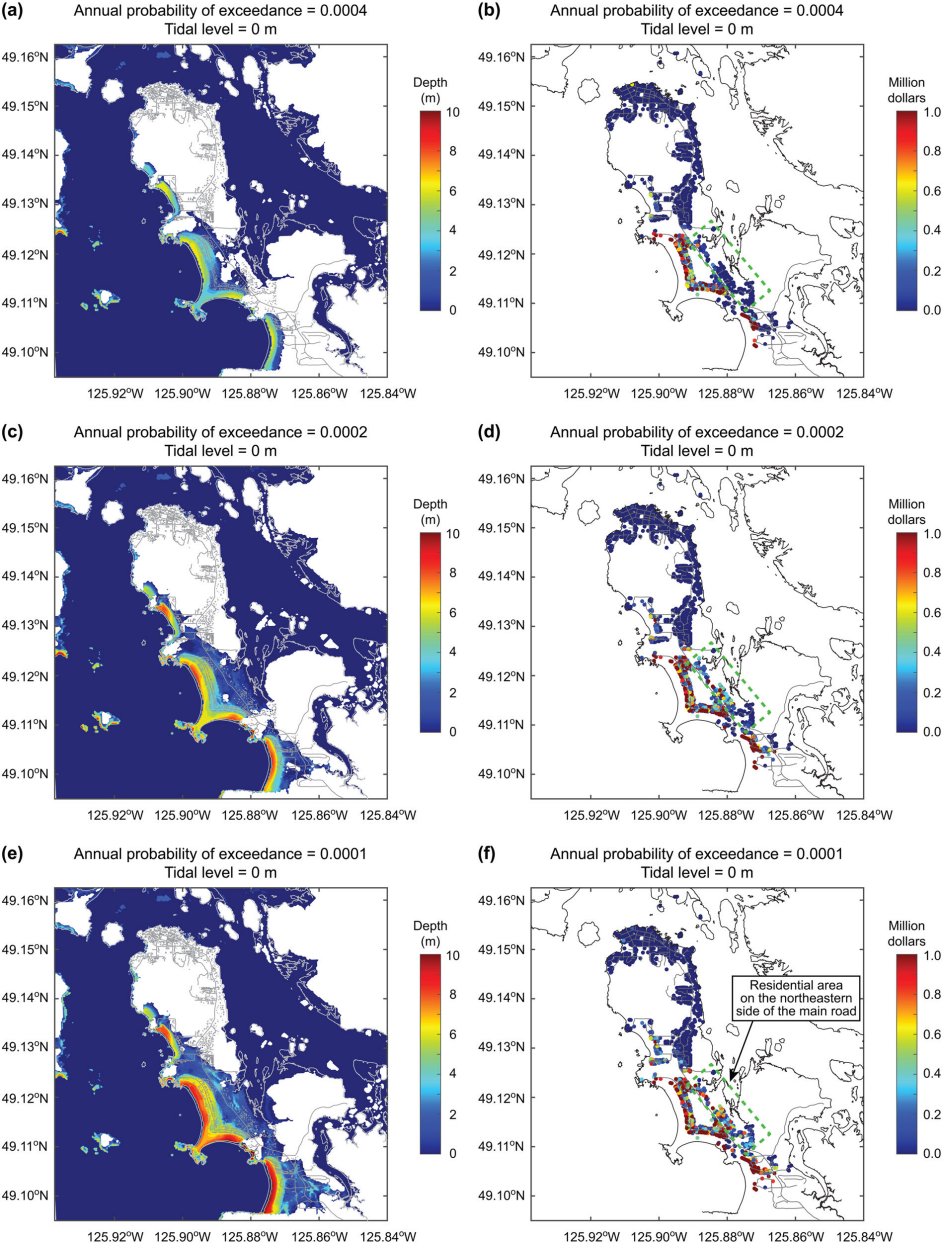
Tsunami inundation maps and tsunami loss maps for different annual probabilities of exceedance. **a** Inundation map for the annual probability of exceedance of 0.0004, **b** loss map for the annual probability of exceedance of 0.0004, **c** inundation map for the annual probability of exceedance of 0.0002, **d** loss map for the annual probability of exceedance of 0.0002, **e** Inundation map for the annual probability of exceedance of 0.0001, and **f** loss map for the annual probability of exceedance of 0.0001. The tidal level is set to 0 m.

From the viewpoint of tsunami risk management, it is crucial to visualize the tsunami inundation corresponding to specific tsunami risk profiles. Since the probabilistic tsunami risk method based on the stochastic source modeling allows to retain information on the contributing earthquake scenarios at different probability levels (see the Method section), tsunami inundation maps and tsunami loss maps for the annual probability of exceedance of 0.0004, 0.0002, and 0.0001 (which approximately corresponds to return periods of 2500, 5000, and 10000 years when expressed in terms of a Poisson process) are provided. The corresponding tsunami loss values are indicated with circle symbols in Fig. 4a. An inspection of the tsunami inundation and loss maps clearly demonstrates that tsunami amplitude and spatial extent become more intense with the decreasing annual probability of exceedance (i.e., more extreme events). For instance, at the annual probability of exceedance = 0.0002, the low-lying part of Tofino will be entirely flooded by the tsunami (Fig. 5c). Some houses on the northeastern side of the main road will be damaged by the tsunamis at the annual probability of exceedance levels of 0.0002 and 0.0001 (Fig. 5d, f; the corresponding residential areas are enclosed by the green broken-line box).

### Time-dependent tsunami loss curves and evolution of tsunami risk metrics

**Fig. 6:**
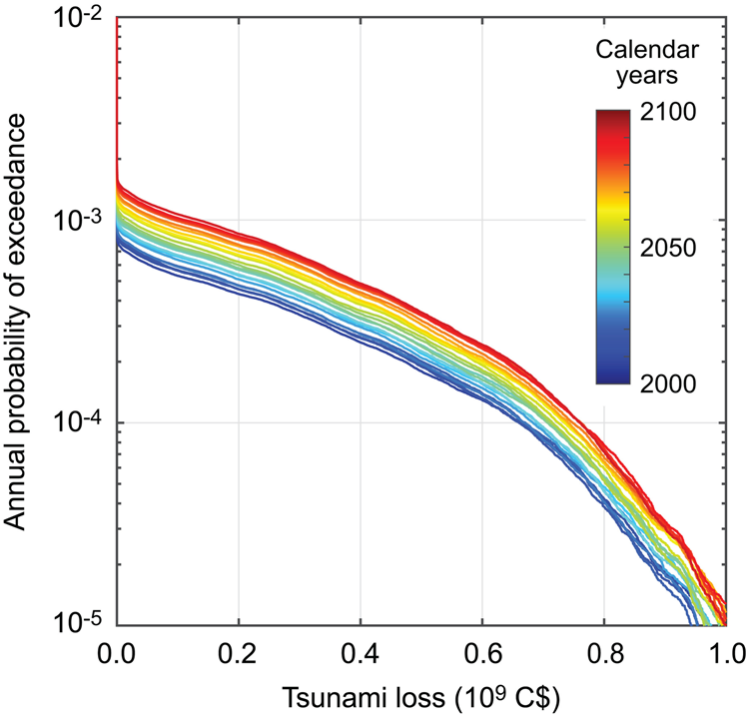
Exceedance probability tsunami loss curves for the time-dependent hazards for different elapsed times since the last major event in 1700. The different colors correspond to different calendar years or elapsed times. The tidal level is set to 0 m.

The time-dependent tsunami risk analysis is iterated by considering a range of the elapsed times since the last major event. To make the analyzed cases relevant to actual tsunami risk management in Tofino, the elapsed time varies from 300 years to 400 years with 5-year intervals corresponding to Year 2000 and Year 2100, respectively (see also Fig. 3). The obtained exceedance probability loss curves are shown in Fig. 6; the curves for different elapsed times or calendar years are displayed with different colors. The results displayed in Fig. 6 demonstrate the evolutionary aspects of tsunami risks over the 21^st^ century.

**Fig. 7:**
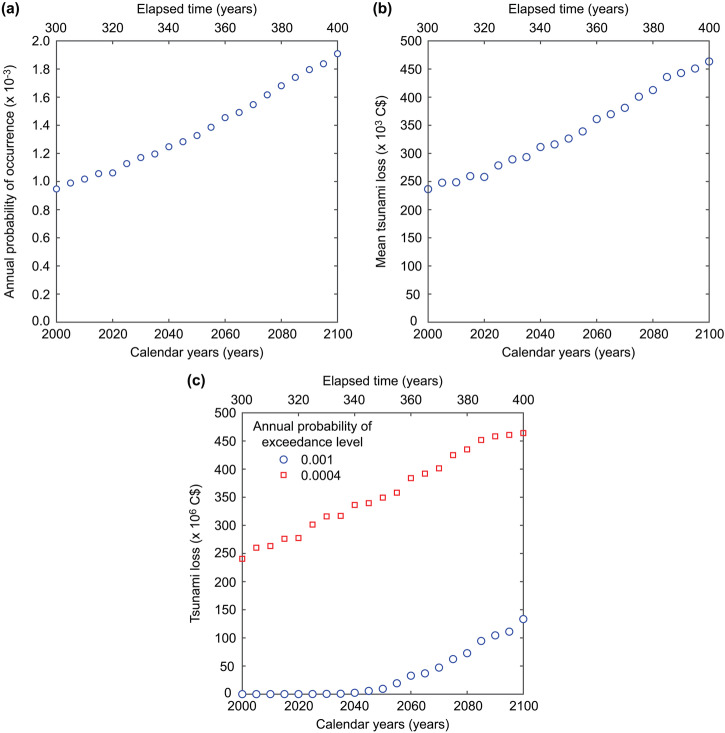
Variations of the tsunami hazard and risk metrics over the calendar years between 2000 and 2100. **a** Annual probability of occurrence, **b** annual average loss, and **c** values at risk for the annual probability of exceedance of 0.001 and 0.0004.

Since various useful tsunami risk metrics can be computed for each elapsed time, the annual probability of tsunami loss occurrence (i.e., an intersection point of a tsunami loss curve and the vertical axis at zero tsunami loss), the mean tsunami loss (i.e., AAL, an area under the EP curve), and tsunami loss values (VaR) at the annual probabilities of exceedance of 0.001 and 0.0004 are extracted and plotted in Fig. 7 as a function of the calendar year or elapsed time. All four tsunami risk metrics monotonically increase with the elapsed time. Between the current time and the Year 2100, the annual probability of tsunami loss occurrence, AAL, and VaR at the annual probability of exceedance of 0.0004 will increase by approximately 80%, and their trend is approximately linear. On the other hand, the increase of the VaR at the annual probability of exceedance of 0.001 is nonlinear because when the elapsed time is not progressed (before 2040), the occurrence of the large tsunami loss event is rare, and thus the tsunami loss is small.

### Effect of tidal level on tsunami loss curves and tsunami inundation-loss maps

The previous results, shown in Fig. 4 to Fig. 7, are conducted by considering the tidal level of 0 m. The tidal records in Tofino show plus and minus 2 m fluctuations with respect to the mean sea level. Also, the most recent predictions of relative sea level rise for Canada^33^ indicate that the sea level in Clayoquot Sound can increase by 0.7 m in 2100 by considering the upper (95^th^ percentile) estimate of the Representative Concentration Pathway (RCP) 8.5 scenario. In the predictions of the relative sea level rise, the vertical ground deformation due to glacial isostatic adjustment in southwestern British Columbia was accounted for, which reduces the original impact of the sea level rise due to meltwater from glaciers, ice caps, and ice sheets. Based on these tidal and sea level rise effects, practically relevant tidal levels for tsunami risk management purposes can be taken as 1 m or 2 m above the current mean sea level.

**Fig. 8:**
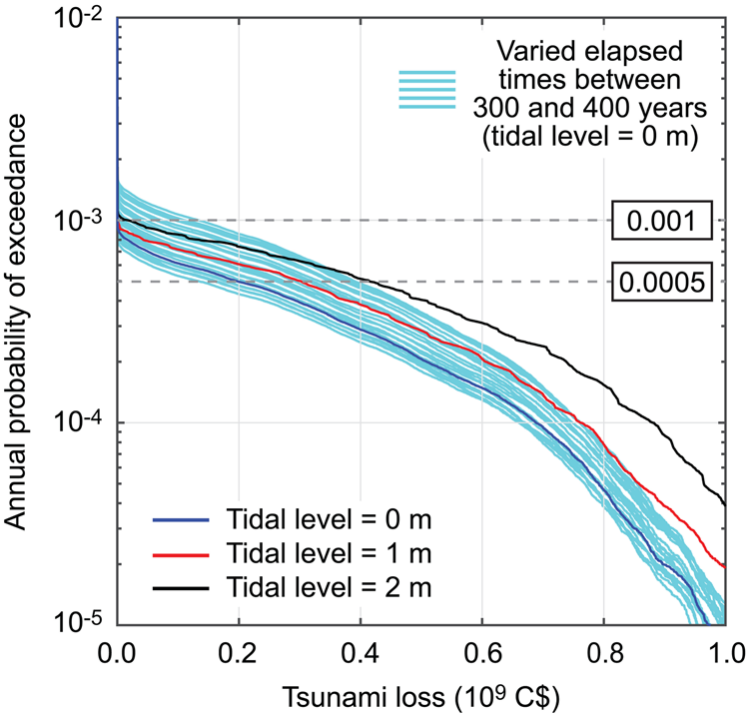
Exceedance probability tsunami loss curves for different tidal levels of 0 m, 1 m, and 2 m. As the benchmark for time-dependent tsunami hazards, a set of exceedance probability tsunami loss curves based on varied elapsed times between 300 and 400 years (Fig. 6) is displayed in the background.

To account for the effects of increased tidal levels on the tsunami inundation hazard and tsunami building damage risk, tsunami simulations are carried out by considering the baseline tidal levels of 1 m and 2 m. Using these results, the tsunami risk assessments are carried out for the elapsed time of 323 years (i.e., current year), and the tsunami loss curves are shown in Fig. 8. As benchmark, the tsunami loss curve for the tidal level = 0 m (as shown in Fig. 4) and the group of tsunami hazard curves based on the elapsed times of 300 years to 400 years (as shown in Fig. 6) are also included in Fig. 8 (see the blue line for the former and cyan lines for the latter). Figure 8 shows the significant impact of the increased baseline sea level on the tsunami loss curves, and their effects are nonlinear, which can be inspected by comparing the differences of the tsunami loss curves between 0 m and 1 m tidal level cases and those between 1 m and 2 m tidal level cases. At the annual probability of exceedance levels near 0.001, the effects of increased baseline sea level have less influence than those due to the varied elapsed times. On the other hand, the coincidence of the major tsunamis (at the annual probability of exceedance levels less than 0.0005) with the 2-m high tides can amplify the anticipated tsunami consequences significantly. However, in interpreting the results presented in Fig. 8, caution is necessary because a tidal level of 2 m is close to the most extreme tidal conditions in Tofino, and the uncertainty of variable tidal levels is not explicitly considered in the presented result (i.e., the tsunami loss curves constitute a conservative upper envelope).

**Fig. 9:**
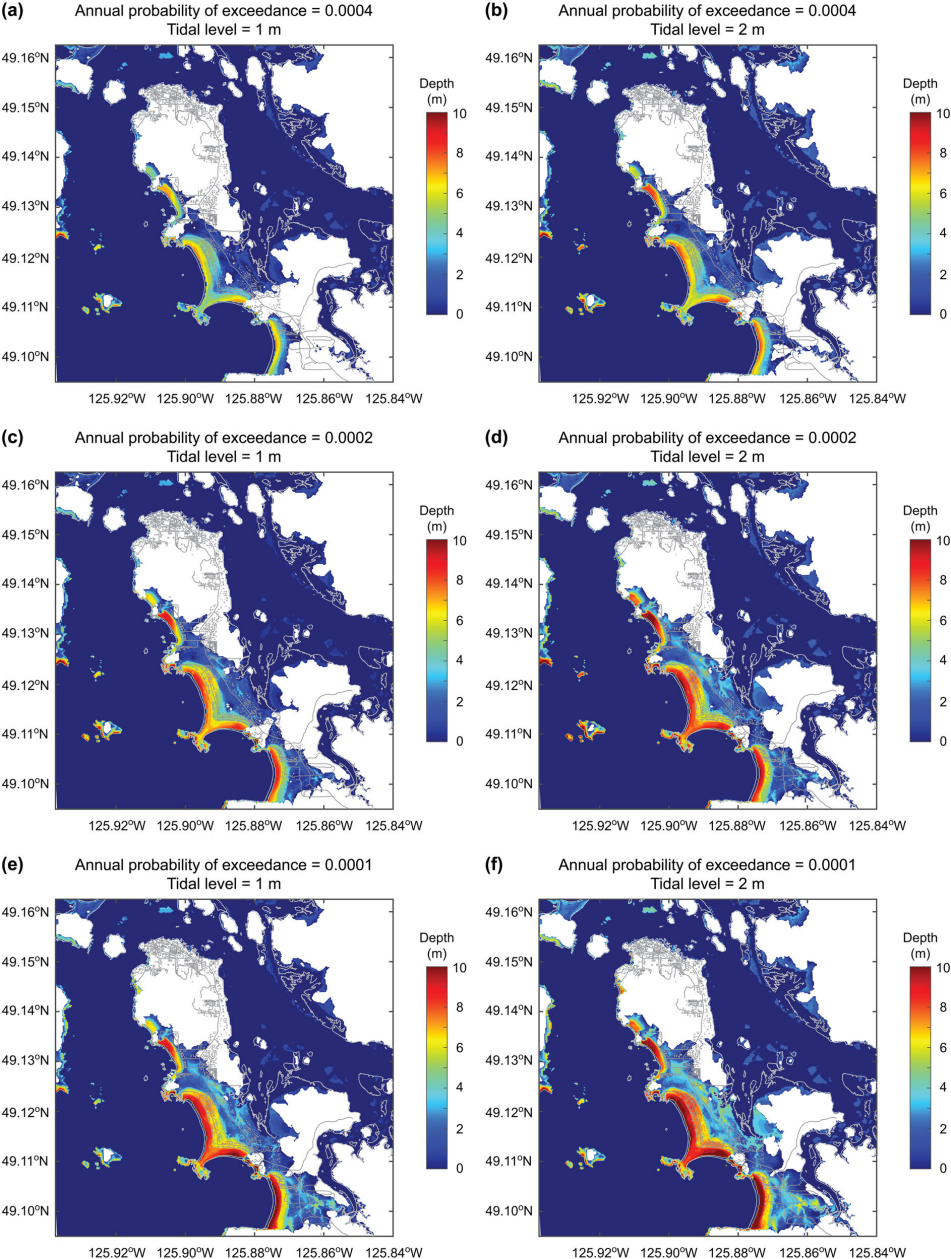
Tsunami inundation maps for different annual probabilities of exceedance by considering different tidal levels above the mean sea level. **a** Annual probability of exceedance = 0.0004 and tide level = 1 m, **b** annual probability of exceedance = 0.0004 and tide level = 2 m, **c** annual probability of exceedance = 0.0002 and tide level = 1 m, **d** annual probability of exceedance = 0.0002 and tide level = 2 m, **e** annual probability of exceedance = 0.0001 and tide level = 1 m, and **f** annual probability of exceedance = 0.0001 and tide level = 2 m.

**Fig. 10:**
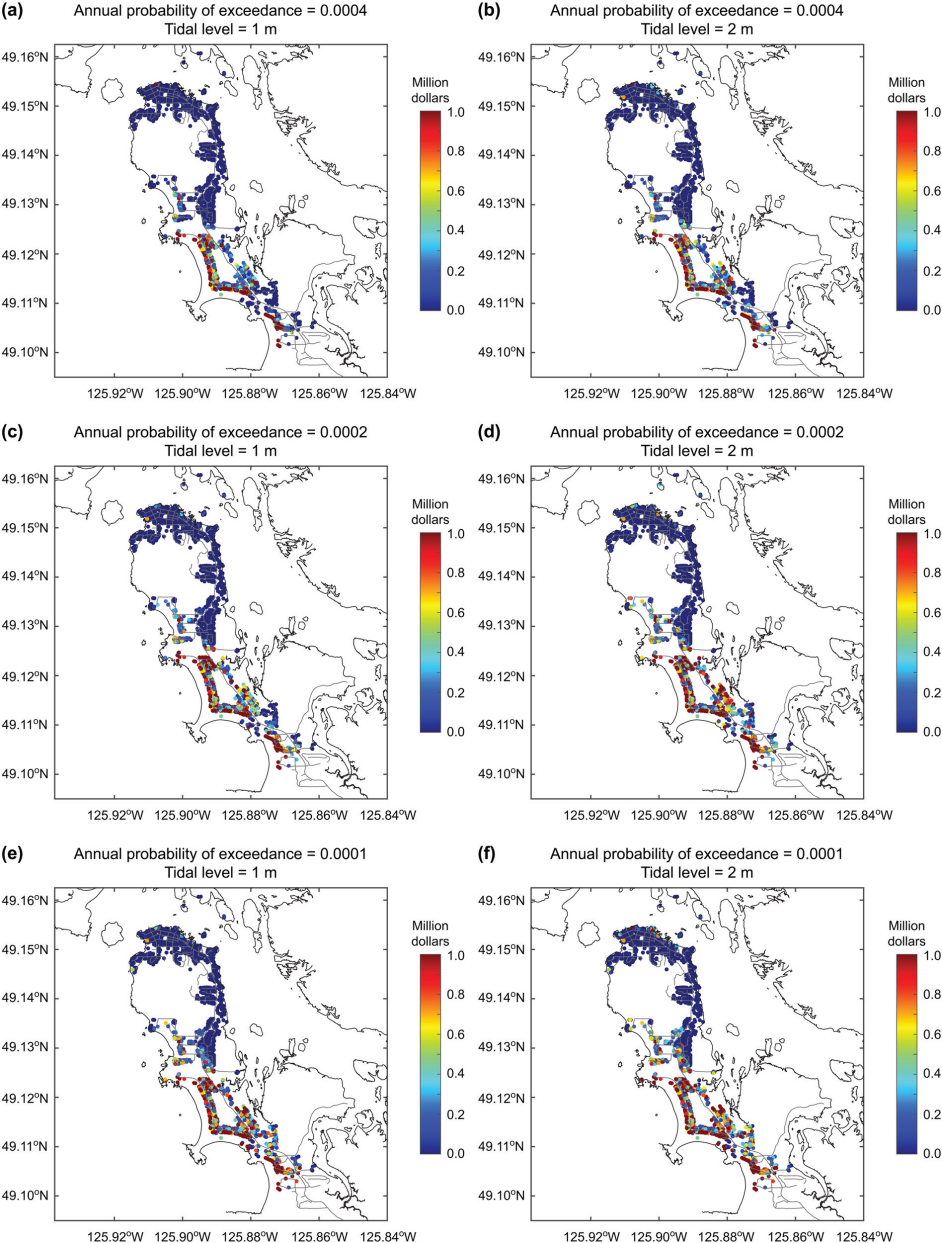
Tsunami loss maps for different annual probabilities of exceedance by considering different tidal levels above the mean sea level. **a** Annual probability of exceedance = 0.0004 and tide level = 1 m, **b** annual probability of exceedance = 0.0004 and tide level = 2 m, **c** annual probability of exceedance = 0.0002 and tide level = 1 m, **d** annual probability of exceedance = 0.0002 and tide level = 2 m, **e** annual probability of exceedance = 0.0001 and tide level = 1 m, and **f** annual probability of exceedance = 0.0001 and tide level = 2 m.

For emergency preparedness purposes, examining the tsunami inundation and loss maps that correspond to the probability levels relevant to tsunami risk management is helpful. Figure 9 shows the tsunami inundation maps corresponding to the annual exceedance probability of 0.0004, 0.0002, and 0.0001 by considering the baseline tidal levels of 1 m and 2 m. In contrast, Fig. 10 shows the tsunami loss maps corresponding to the annual exceedance probability of 0.0004, 0.0002, and 0.0001 by considering the baseline tidal levels of 1 m and 2 m. The intensification of tsunami inundation and resulting tsunami loss due to the higher baseline sea levels can be observed by comparing the tsunami inundation and loss maps shown in Figs. 9 and 10 with those shown in Fig. 7, highlighting significant tsunami risks in the low-lying part of Tofino.

## Discussion

Risk quantification is essential for making informed decisions regarding tsunami risk mitigation by considering critical uncertainties of the underlying hazard, exposure, and vulnerability components. The current tsunami risk assessment approaches require extending long-term hazard and risk assessments with time-dependent or non-stationary phenomena. This study contributed to the existing literature by developing a novel time-dependent tsunami risk model for the coastal town of Tofino subjected to the major tsunami threats from the Cascadia subduction earthquakes. The innovative aspects of the developed tsunami risk model include the time-dependent earthquake occurrence model that reflects the uncertainty of the underlying geological data, heterogeneous earthquake slip distributions with variable fault-plane geometry, high-resolution bathymetry-elevation data of local topography, and high-quality building inventory data. Tofino’s evolutionary tsunami risk profiles over the 21^st^ century underscore the importance of considering time-dependent earthquake hazards for future tsunami risk assessments. Moreover, uncertainty associated with baseline sea level significantly influences tsunami risk assessments, and their effects are nonlinear.

The tsunami risk results for Tofino offer valuable insights related to tsunami risk management and emergency preparedness by local municipalities and governments. Extreme tsunami inundation situations, which are displayed in Figs. 7, 9, and 10, reveal a serious challenge for tsunami evacuation in Tofino, where people who are residing and staying along the beaches will need to travel relatively far distances to reach high grounds (above 20 m in elevation). Such evacuation may take longer than the tsunami arrival time in Tofino^34^. There is a need to create a vertical evacuation shelter in the low-lying part of Tofino and to improve the evacuation routes^35,36^.

The time-dependent tsunami risk model can be further improved by accounting for variable tidal levels and relative sea level changes. For instance, tidal variations can be characterized using tidal records in Tofino, and non-stationary and uncertain relative sea level rises at future times can be directly incorporated into the tsunami risk assessments by using multiple RCP scenarios at different future times (e.g., nine predictions of relative sea level rise for Canada^33^ by considering three RCP scenarios (2.6, 4.5, and 8.5) and three percentile levels (median, 5^th^, and 95^th^)).

Another critical non-stationary aspect of the tsunami risk for Tofino, which is not reflected in the current study, is the changing exposure characteristics. The population in Tofino has increased from approximately 500 in the early 1970s, to about 1000 in 1990 and 2500 as of 2021. In the past decade, the population has increased with annual growth rates between 2% and 3%. This trend will continue, and more buildings will be constructed. Consequently, more population will be exposed to tsunami threats. It is also essential to be aware that the increased population, when the available habitable land is limited (like Tofino), can stress the existing infrastructure, such as roads and water supply systems. The criticality of infrastructure becomes more important in a situation of catastrophic events, such as mega-tsunamis.

Finally, the current risk assessment for Tofino is concerned only with tsunami risks. However, the Cascadia megathrust earthquakes inevitably cause significant shaking in Tofino, followed by aftershocks^37^. In the long term, the megathrust earthquakes will cause subsidence of the ground in Tofino, which exacerbates the hydrometeorological impacts due to storms and coastal flooding. Future disaster risk impact assessments should address the issues related to cascading and compounding risks by considering hazard interaction and dynamic vulnerability in the multi-hazard context^38,39^.

## Methods

### **Earthquake occurrence and magnitude model (**Fig. 2a**)**

The renewal model for the full rupture of the Cascadia subduction earthquakes^29^ is developed using offshore turbidite records^23^. The turbidite data identified 40 events over the last 10,000 years through synchronous analysis of offshore geological cores along the coastline of the Pacific Northwest. Nineteen of the identified events ruptured the entire length of the Cascadia subduction zone, whereas the remaining events ruptured the middle and southern parts of the subduction zone only (i.e., Oregon and northern California). To account for the inherent uncertainty of the offshore turbidite data and histories, Monte Carlo resampling of the Cascadia age data was carried out to characterize the inter-arrival times of the whole margin Cascadia subduction events^29^. Compared with the Gaussian distribution, the resampled inter-arrival time data exhibit heavier tails on both upper and lower sides. The mean and standard deviation of the simulated inter-arrival time data are 561 years and 272 years, respectively. To model the inter-arrival time data for the full-rupture Cascadia subduction events, the 3-component Gaussian mixture model was used^29^. In the 3-component Gaussian mixture model, the mixing proportions of the three Gaussian components can be adjusted when the elapsed time since the last event needs to be considered (Fig. 3). Note that parameters of the 3-component Gaussian mixture model were estimated by using only closed inter-arrival time data. The inclusion of open inter-arrival time data can have influence on the estimated parameters^40^. In addition, the distribution type of the mixture model could be changed to distributions that only take positive values. In the future, alternative resampling approaches apart from the adopted one^30^ and more rigorous statistical investigations can be performed to improve the Gaussian mixture model considered in this study. Such earthquake occurrence models can also be implemented within a Bayesian updating scheme^41^.

Moreover, it is important to clarify that the interpretations of the rupture history of the Cascadia subduction events that are adopted in developing the Gaussian mixture model are not unique. Earthquake occurrence models can be developed by adopting alternative hypotheses and interpretations^42,43^ and such models can be implemented in a logic tree to capture this uncertainty in probabilistic tsunami hazard and risk assessments.

The earthquake magnitude distribution is critical for tsunami hazard assessments. The magnitudes of the Cascadia subduction events primarily depend on the rupture patterns and corresponding rupture areas. In the developed tsunami risk model, two end-member magnitude models are considered by assigning equal weights to the two models. The first one is the Gutenberg–Richter model with the *b*-value of 1. The second one is based on the characteristic magnitude model with the uniform distribution. Since the whole rupture scenarios are concerned, the minimum and maximum magnitudes are set to 8.7 and 9.1 for both magnitude models. The earthquake occurrence models control the occurrence probabilities of the full-margin megathrust Cascadia events. The uncertainty characterization of the earthquake magnitude distribution can have a significant influence on the tsunami hazard assessments; therefore, the future study should explore a wider range of the distributions and their parameters.

### **Stochastic rupture model (**Fig. 2b**)**

The fault plane geometry for the Cascadia subduction zone is based on the Slab2 model^44^. This fault plane is approximated by a set of 7,452 sub-faults that reach depths of 30 km, each having a size of 5.6 km along strike and 3.8 km along dip. To simulate an earthquake slip distribution as random realization, a scenario magnitude is specified with a 0.1 magnitude bin. The magnitude value is simulated from the uniform distribution within the magnitude bin. Subsequently, eight earthquake source parameters, i.e., fault length, fault width, mean slip, maximum slip, Box-Cox parameter, along-strike correlation length, along-dip correlation length, and Hurst number, are generated from the scaling relationships^45^. Once a suitable fault geometry is determined, the fault plane is placed randomly within the Cascadia zone. It is noted that the scaling relationships for the fault geometry and the earthquake slip statistics^45^ are based on the assumption of constant rock rigidity (as adopted by the underlying source inversion studies).

A heterogeneous earthquake slip distribution is generated for a given fault plane geometry. A trial slip distribution is first simulated from an anisotropic von Kármán wavenumber spectrum with its amplitude spectrum being parametrized by along-strike correlation length, along-dip correlation length, and Hurst number and its phase being randomly distributed between 0 and 2π^46^. The simulated slip distribution is modified via Box-Cox power transformation to achieve a desirable right-skewed feature of the marginal distribution of earthquake slip over the fault plane^45^. To ensure that the simulated earthquake slip distribution has realistic characteristics for the target Cascadia events, major asperities are constrained to occur in the shallow part of the subduction interface (the lower depth limit is set to 13 km^27^) to coincide broadly with the outer wedge of the accretionary prism^47^. If the trial slip distribution does not meet the criteria, this realization is discarded, and another trial model is generated. This process is continued until an acceptable model is obtained. By repeating the above procedure 500 times for each of the ten bins between *M*_w_ 8.1 and *M*_w_ 9.1, a set of 5,000 earthquake rupture models is generated. Note that out of the generated 5,000 rupture models, only 2000 full-margin rupture models having earthquake magnitudes between *M*_w_ 8.7 and *M*_w_ 9.1 are considered in the tsunami hazard and risk assessments. The stochastic rupture models can represent different fault geometry, positions within the overall fault plane of the Cascadia subduction zone, and heterogeneous earthquake slip distributions.

### **Tsunami inundation model (**Fig. 2c**)**

To evaluate tsunami inundations in Tofino by considering numerous stochastic earthquake rupture models, ground deformations due to earthquake ruptures are calculated^48,49^, and then nonlinear shallow water equations are solved using the TUNAMI code^50^. For this purpose, nested grids of 810-m, 270-m, 90-m, 30-m, 10-m, and 5-m that cover the entire Cascadia subduction zone are set up by combining global bathymetry data (GEBCO–450-m; https://www.gebco.net/), national bathymetry data (CHS–10-m; https://open.canada.ca/data/en/dataset/d3881c4c-650d-4070-bf9b-1e00aabf0a1d), national elevation data (CDEM–20-m; https://open.canada.ca/data/en/dataset/7f245e4d-76c2-4caa-951a-45d1d2051333), and LiDAR-derived topographic contours (0.5-m; https://lidar.gov.bc.ca/). The high-resolution local elevation data cover the District of Tofino (Fig. 1b). The graphical presentation of the nested grid system can be found in the published study^31^. The vertical reference datum is at the mean sea level. For all computational cells, the bottom friction and surface roughness are represented by a Manning’s coefficient equal to 0.025 m^−1/3^s, which is often used for agricultural land and ocean/water. It is noted that the adopted Manning’s coefficient value of 0.025 m^−1/3^s is relatively low, compared to those for built-up areas^51^. Therefore, the calculated tsunami inundation extent may be overestimated from this perspective. The run-up calculation is determined by evaluating a dry/wet condition of a computational cell based on total water depth relative to its elevation. Moreover, the effects of coseismic ground deformation are considered by adjusting the elevation data prior to the tsunami simulation run.

Each tsunami simulation is performed for 2 hours, sufficient to model the most critical phase of tsunami waves for the Cascadia tsunami scenarios. Due to the strong directivity of radiated tsunami waves, earthquake rupture scenarios that rupture the central and southern margins only (i.e., not rupturing the northern margin off Vancouver Island) do not generate large tsunami waves at Tofino^27^ (typically, less than 1 m in terms of maximum wave amplitude above the mean sea level). For this reason, the high-resolution tsunami inundation simulations using 5-m grids are performed for 2,000 stochastic source scenarios with earthquake magnitudes between *M*_w_ 8.7 and *M*_w_ 9.1. Regarding the number of tsunami simulation runs, the stability of the probability distribution functions of tsunami hazard and risk metrics of interest (e.g., offshore tsunami wave height, inundation area, and tsunami loss for Tofino) has been ensured for each magnitude bin. Such results for convergence check can be found in the previous tsunami risk study for Tofino^31^. Moreover, the variable tidal levels can have significant impacts on the inundation results^52^, therefore the aspects should be improved in the future.

### **Building and infrastructure exposure model (**Fig. 2d**)**

To carry out earthquake-tsunami risk assessments, the District of Tofino has developed a comprehensive inventory of buildings. Most buildings in Tofino Town are at relatively high elevations (typically above 10 m) and are protected from the direct tsunami waves, whereas buildings along the McKenzie, Chesterman, and Cox Bay beaches are at low elevations and open to the Pacific (Fig. 1b). The building data are related to the building classification scheme adopted by the Natural Resources Canada^53^. In the developed tsunami risk model, a portfolio of 1,789 buildings is considered, excluding campsites, marina docks, and non-permanent buildings. Most buildings are 1- to 2-story wooden houses constructed in the 1960s or afterwards. The total replacement cost of a building, which consists of structural elements, non-structural elements, and building contents, is typically less than C$ 2 million, with an average value of C$ 1.27 million and a total asset value of C$ 2.27 billion.

### **Tsunami fragility model (**Fig. 2e**)**

The empirical tsunami fragility model^19^ is adopted to evaluate the extent of tsunami damage to buildings in Tofino. The model was developed based on the tsunami damage data from the 2011 Tohoku event in Japan, containing more than 200,000 observations. This choice is due to the unavailability of local/regional tsunami fragility models for southwestern British Columbia and the strong preference for empirical tsunami damage data. The tsunami fragility model is characterized using multinomial logistic regression analysis by considering the structural typology (i.e., wood, concrete, steel, and masonry and others), number of stories, and topographical indicators (i.e., coastal plain and ria) as explanatory variables. Figure 2e shows tsunami fragility functions of wooden buildings for five tsunami damage levels, i.e., minor, moderate, extensive, complete, and collapse.

### **Tsunami damage and loss estimation (**Fig. 2f**)**

For tsunami loss estimation, these tsunami damage levels, identified using the tsunami fragility functions, are related to building damage ratio ranges of 0.03–0.1, 0.1–0.3, 0.3–0.5, 0.5–1.0, and 1.0, respectively (http://www.mlit.go.jp/toshi/toshi-hukkou-arkaibu.html). During the tsunami damage and loss analyses, the tsunami fragility functions are applied using flow depth values at individual buildings from tsunami inundation simulations. The damage states are assigned probabilistically by comparing a uniform random number between 0 and 1 with the corresponding tsunami damage probabilities. If the random number falls within the range of tsunami damage probabilities for a specific damage level, the tsunami damage state is selected, and the tsunami damage ratio is subsequently sampled within the suggested range. Finally, the tsunami loss value is determined by multiplying the total asset value of the property and the sampled damage ratio. The above procedure is repeated for all buildings for a given inundation scenario.

### Probabilistic tsunami risk assessment

To perform a time-dependent tsunami risk assessment for the building portfolio of interest, Monte Carlo simulations are implemented by generating numerous stochastic event sets, each with a specified duration. The duration of each stochastic event set is set to 1 year, while the simulated number of stochastic event sets is set to 10 million. It is noted that the majority of the stochastic event sets do not contain any Cascadia subduction events because of their low probability of occurrence (on average 1 in 561 chance). The stochastic event sets reflect the time-dependency of the Cascadia subduction events and depend on the time that has elapsed since the last major event. The tsunami event is assigned from the stochastic source models for each generated Cascadia event in the stochastic event set. The tsunami inundation depths at the building locations are further perturbed by considering a multiplicative factor that is modeled by the lognormal distribution with a median of 1 and a coefficient of variation of 0.1. This multiplicative factor is incorporated to capture the unmodeled aspects of tsunami inundation simulations in relation to the comparison between the historical inundation observations and the modeled results^16^. The consideration of the multiplicative factor increases the variability of the tsunami risk assessment slightly and makes the exceedance probability tsunami loss curve smoother. Subsequently, the tsunami damage-loss estimation is performed using the building exposure model, tsunami fragility function, and tsunami damage-loss ratios. The final outputs from the probabilistic tsunami risk model are the 10 million tsunami loss samples, each corresponding to 1-year duration. Using the simulated loss data, the exceedance probability tsunami loss curve can be derived, and the corresponding tsunami risk metrics can be obtained.

## Data Availability

No datasets were generated or analyzed during the current study.

## References

[1] Mori N (2022). Giant tsunami monitoring, early warning and hazard assessment. Nat. Rev. Earth Environ..

[2] Wald DJ, Franco G (2016). Money matters: rapid post-earthquake financial decision-making. Nat. Hazards Observer.

[3] Cosson C (2020). Build Back Better: between public policy and local implementation, the challenges in Tohoku’s reconstruction. Architect. Urban Plan.

[4] Kagan Y, Jackson DD (2013). Tohoku earthquake: a surprise?. Bull. Seismol. Soc. Am..

[5] Geist EL, Parsons T (2006). Probabilistic analysis of tsunami hazards. Nat. Hazard..

[6] Thio HK, Somerville P, Ichinose G (2007). Probabilistic analysis of strong ground motion and tsunami hazards in Southeast Asia. J. Earthq. Tsunami.

[7] Grezio A, Tonini R, Sandri L, Pierdominici S, Selva J (2015). A methodology for a comprehensive probabilistic tsunami hazard assessment: multiple sources and short-term interactions. J. Mar. Sci. Eng..

[8] Mitchell-Wallace, K., Jones, M., Hillier, J. & Foote, M. *Natural Catastrophe Risk Management and Modelling: A Practitioner’s Guide*. Wiley-Blackwell: Chichester, United Kingdom, 536 p. (2017).

[9] Behrens J (2021). Probabilistic tsunami hazard and risk analysis: a review of research gaps. Front. Earth Sci..

[10] Goda K, De Risi R (2017). Probabilistic tsunami loss estimation: stochastic earthquake scenario approach. Earthq. Spectra.

[11] Park H, Cox DT, Barbosa AR (2017). Comparison of inundation depth and momentum flux based fragilities for probabilistic tsunami damage assessment and uncertainty analysis. Coast. Eng..

[12] Fukutani Y, Moriguchi S, Terada K, Otake Y (2021). Time-dependent probabilistic tsunami inundation assessment using mode decomposition to assess uncertainty for an earthquake scenario. J. Geophys. Res.: Oceans.

[13] Alhamid AK, Akiyama M, Aoki K, Koshimura S, Frangopol DM (2022). Stochastic renewal process model of time-variant tsunami hazard assessment under nonstationary effects of sea-level rise due to climate change. Struct. Saf..

[14] Li L (2016). How heterogeneous coseismic slip affects regional probabilistic tsunami hazard assessment: a case study in the South China Sea. J. Geophys. Res.: Solid Earth.

[15] Melgar D, Williamson AL, Salazar-Monroy EF (2019). Differences between heterogenous and homogenous slip in regional tsunami hazards modelling. Geophys. J. Int..

[16] Fukutani Y, Suppasri A, Imamura F (2015). Stochastic analysis and uncertainty assessment of tsunami wave height using a random source parameter model that targets a Tohoku-type earthquake fault. Stochastic Environ. Res. Risk Assess..

[17] Miyashita T, Mori N, Goda K (2020). Uncertainty of probabilistic tsunami hazard assessment of Zihuatanejo (Mexico) due to the representation of tsunami variability. Coast. Eng. J..

[18] Suppasri A (2013). Building damage characteristics based on surveyed data and fragility curves of the 2011 Great East Japan tsunami. Nat. Hazards.

[19] De Risi R, Goda K, Yasuda T, Mori N (2017). Is flow velocity important in tsunami empirical fragility modeling?. Earth-Sci. Rev..

[20] Hyndman RD, Rogers GC (2010). Great earthquakes on Canada’s west coast: a review. Can. J. Earth Sci..

[21] AECOM Modeling of potential tsunami inundation limits and run-up. Report for the Capital Region District. Available online: https://www.crd.bc.ca/docs/default-source/news-pdf/2013/modelling-of-potential-tsunami-inundation-limits-and-run-up-report-.pdf (2013).

[22] Atwater, B. & Hemphill-Haley, E. *Recurrence intervals for great earthquakes of the Past 3500 years at Northeastern Willapa Bay, Washington*. United States Geological Survey. Professional Paper 1576. (1997). Available online: https://pubs.er.usgs.gov/publication/pp1576.

[23] Goldfinger, C. et al. *Turbidite event history: methods and implications for Holocene paleoseismicity of the Cascadia subduction zone*. United States Geological Survey Professional Paper 1661–F (2012). Available online: https://pubs.er.usgs.gov/publication/pp1661F.

[24] Satake K, Shimazaki K, Tsuji Y, Ueda K (1996). Time and size of a giant earthquake in Cascadia inferred from Japanese tsunami records of January 1700. Nature.

[25] Takabatake T, St‑Germain P, Nistor I, Stolle J, Shibayama T (2019). Numerical modelling of coastal inundation from Cascadia subduction zone tsunamis and implications for coastal communities on Western Vancouver Island, Canada. Nat. Hazard..

[26] Gao D (2018). Defining megathrust tsunami source scenarios for northernmost Cascadia. Nat. Hazard..

[27] Goda K (2022). Stochastic source modelling and tsunami simulations of Cascadia subduction earthquakes for Canadian Pacific coast. Coast. Eng. J..

[28] Goda K (2023). Probabilistic tsunami hazard analysis for Vancouver Island coast using stochastic rupture models for the Cascadia subduction earthquakes. GeoHazards.

[29] Goda K (2023). Statistical modeling of full-margin rupture recurrence for Cascadia subduction zone using event time resampling and Gaussian mixture method. Geosci. Lett..

[30] Kulkarni R, Wong I, Zachariasen J, Goldfinger C, Lawrence M (2013). Statistical analyses of great earthquake recurrence along the Cascadia subduction zone. Bull. Seismol. Soc. Am..

[31] Goda K, Orchiston K, Borozan J, Novakovic M, Yenier E (2023). Evaluation of reduced computational approaches to assessment of tsunami hazard and loss using stochastic source models: case study for Tofino, British Columbia, Canada, subjected to Cascadia megathrust earthquakes. Earthq. Spectra.

[32] Walton M (2021). Toward an integrative geological and geophysical view of Cascadia Subduction Zone earthquakes. Ann. Rev. Earth Planet. Sci..

[33] James, T. S., Robin, C., Henton, J. A. & Craymer, M. *Relative sea-level projections for Canada based on the IPCC Fifth Assessment Report and the NAD83v70VG national crustal velocity model*. Geological Survey of Canada, Open File 8764. 10.4095/327878 (2021).

[34] Takabatake T, Nistor I, St-Germain P (2020). Tsunami evacuation simulation for the District of Tofino, Vancouver Island, Canada. Int. J. Disaster Risk Reduct..

[35] Mostafizi A, Wang H, Cox D, Dong S (2019). An agent-based vertical evacuation model for a near-field tsunami: choice behavior, logical shelter locations, and life safety. Int. J. Disaster Risk Reduct..

[36] Muhammad A (2021). Are current tsunami evacuation approaches safe enough?. Stochastic Environ. Res. Risk Assess..

[37] Zhang L, Werner MJ, Goda K (2020). Stability of ETAS parameters in global subduction zones and applications to mainshock-aftershock hazard assessment. Bull. Seismol. Soc. Am..

[38] Gill JC, Malamud BD (2016). Hazard interactions and interaction networks (cascades) within multi-hazard methodologies. Earth Syst. Dyn..

[39] De Risi R, Muhammad A, De Luca F, Goda K, Mori N (2022). Dynamic risk framework for cascading compounding climate-geological hazards: a perspective on coastal communities in subduction zones. Front. Earth Sci..

[40] Parsons, T. Monte Carlo method for determining earthquake recurrence parameters from short paleoseismic catalogs: example calculations for California. *J. Geophys. Res.: Solid Earth*, 113. 10.1029/2007JB004998. (2008).

[41] Grezio A, Marzocchi W, Sandri L, Gasparini P (2010). A Bayesian procedure for Probabilistic Tsunami Hazard Assessment. Natural Hazards.

[42] Atwater BF, Carson B, Griggs GB, Johnson HP, Salmi MS (2014). Rethinking turbidite paleoseismology along the Cascadia subduction zone. Geology.

[43] Hill JC, Watt JT, Brothers DS (2022). Mass wasting along the Cascadia subduction zone: Implications for abyssal turbidite sources and the earthquake record. Earth Planet. Sci. Lett..

[44] Hayes GP (2018). Slab2, a comprehensive sub-duction zone geometry model. Science.

[45] Goda K, Yasuda T, Mori N, Maruyama T (2016). New scaling relationships of earthquake source parameters for stochastic tsunami simulation. Coastal Eng. J..

[46] Mai PM, Beroza GC (2002). A spatial random field model to characterize complexity in earthquake slip. J. Geophys. Res.: Solid Earth.

[47] Watt JT, Brothers DS (2021). Systematic characterization of morphotectonic variability along the Cascadia convergent margin: Implications for shallow megathrust behavior and tsunami hazards. Geosphere.

[48] Okada Y (1985). Surface deformation due to shear and tensile faults in a half-space. Bull. Seismol. Soc. Am..

[49] Tanioka Y, Satake K (1996). Tsunami generation by horizontal displacement of ocean bottom. Geophys. Res. Lett..

[50] Goto, C., Ogawa, Y. Shuto, N. & Imamura, F. Numerical method of tsunami simulation with the leap-frog scheme. IOC Manual 35. UNESCO: Paris, France. (1997).

[51] Kaiser G (2011). The influence of land cover roughness on the results of high resolution tsunami inundation modeling. Nat. Hazard. Earth Syst. Sci..

[52] Adams LM, LeVeque RJ, González FI (2015). The pattern method for incorporating tidal uncertainty into probabilistic tsunami hazard assessment (PTHA). Nat. Hazard..

[53] Hobbs, T., Journeay, J. M. & LeSueur, P. *Developing a retrofit scheme for Canada’s Seismic Risk Model*. Geological Survey of Canada Open File 8822, 10 p. 10.4095/328860 (2021).

